# Frequency Matrix Approach Demonstrates High Sequence Quality in Avian BARCODEs and Highlights Cryptic Pseudogenes

**DOI:** 10.1371/journal.pone.0043992

**Published:** 2012-08-27

**Authors:** Mark Y. Stoeckle, Kevin C. R. Kerr

**Affiliations:** 1 Program for the Human Environment, Rockefeller University, New York, New York, United States of America; 2 Royal Ontario Museum, Department of Natural History, Toronto, Ontario, Canada; UT MD Anderson Cancer Center, United States of America

## Abstract

The accuracy of DNA barcode databases is critical for research and practical applications. Here we apply a frequency matrix to assess sequencing errors in a very large set of avian BARCODEs. Using 11,000 sequences from 2,700 bird species, we show most avian cytochrome c oxidase I (COI) nucleotide and amino acid sequences vary within a narrow range. Except for third codon positions, nearly all (96%) sites were highly conserved or limited to two nucleotides or two amino acids. A large number of positions had very low frequency variants present in single individuals of a species; these were strongly concentrated at the ends of the barcode segment, consistent with sequencing error. In addition, a small fraction (0.1%) of BARCODEs had multiple very low frequency variants shared among individuals of a species; these were found to represent overlooked cryptic pseudogenes lacking stop codons. The calculated upper limit of sequencing error was 8×10^−5^ errors/nucleotide, which was relatively high for direct Sanger sequencing of amplified DNA, but unlikely to compromise species identification. Our results confirm the high quality of the avian BARCODE database and demonstrate significant quality improvement in avian COI records deposited in GenBank over the past decade. This approach has potential application for genetic database quality control, discovery of cryptic pseudogenes, and studies of low-level genetic variation.

## Introduction

Beginning in 2003, researchers have been building a library of short genetic identifiers – DNA barcodes – for all animal, plant, and fungal species [1], [2]. The effort aims to simplify species identification, including for specimens missing diagnostic features (e.g. fragments and immature or vegetative forms) or when taxonomic expertise is not available [3]. The agreed upon standard DNA barcode for animals is a 648 base pair (bp) region encompassing 216 codons of cytochrome *c* oxidase I (COI), which contains enough sequence diversity to separate most species and is relatively easy to amplify from most taxa using a limited set of primers [4]–[6].

Most DNA barcode studies so far focus on diagnostic accuracy in distinguishing closely related species and the biological meaning of discordance, i.e., barcode clusters with multiple species or species with multiple barcode clusters [7]–[11]. Beyond species identification, growing libraries of DNA barcodes offer opportunities for investigating mitochondrial evolution and higher-level taxonomy [12], [13]. COI barcodes represent the largest, most taxonomically diverse set of mitochondrial sequences presently available, with approximately 260,000 records from 37,000 animal species in GenBank under keyword BARCODE. The next largest set of mtDNA sequences in GenBank is cytochrome *b* with 157,000 records from 26,000 species. Advantages of the BARCODE standard include a minimum of 500 bp from a defined region, linkage to museum specimens, and publicly archived trace files documenting a minimum quality score [4].

The accuracy of the barcode reference database is critical to research and practical applications. Potential inaccuracies include incorrect taxonomic labels, overlooked pseudogenes, and sequencing errors. Taxonomic mislabelings due to misidentified specimens, outdated taxonomy, database errors, or laboratory mix-ups are a recognized hazard in nucleotide sequence databases [14]–[16]. The BARCODE standard mandates linkage to museum specimens, helping ensure valid identifications and facilitating re-examination in questionable cases. To minimize depositing mislabeled records, Barcode of Life Datasystems (BOLD) workbench tools highlight sequences with anomalous taxonomic placements in neighbor-joining (NJ) trees and flag records containing stop codons, typically present in pseudogenes [2]. To minimize sequencing errors, the BARCODE standard calls for bidirectional sequencing and publicly archived trace files with minimum PHRED scores. However to date there is no direct way of assessing sequence errors in published records.

Here we test the hypothesis that sequencing errors in reference barcodes can be detected as very low frequency variants at positions that are otherwise highly conserved. We use this approach to assess sequencing error in the recently available, very large avian BARCODE dataset. With GenBank COI records for approximately one-third of the 10,000 species of birds, they are one of the best-sampled animal groups to date [17]–[24]. We suggest application of our findings to quality assessment of nucleotide databases, including a method for identifying cryptic pseudogenes, and discuss implications for studies of low-level sequence variation.

## Methods

Avian BARCODE records in GenBank on January 28, 2012 were retrieved using search phrase: “aves [organism] AND BARCODE [keyword] AND (COI [gene name] OR cox1 [gene name]) NOT phase_0.” “Phase 0” refers to GenBank BARCODE records that are identified only to order; these were excluded from analysis. The resulting fasta file contained 11,333 records with 2,718 species names. Fasta file names were reconciled with an authority file used for All Birds Barcoding Initiative (ABBI) using Name_Lookup available at www.barcodingbirds.org. Twelve synonyms were found, resolving the file into 2,706 species. Taxonomic coverage was assessed via comparison to the ABBI authority file. Sequences were aligned in MEGA using MUSCLE and the resulting alignment was checked by eye [25], [26]. The file was trimmed to include 648 positions corresponding to bovine COI nucleotides 51–699 [4]. To assess changes in record quality over time, a similar procedure was followed except that the publication date field [PDAT] was used to download avian BARCODE and non-BARCODE (“NOT BARCODE [keyword]”) COI records according to date deposited in GenBank, beginning with January 1, 2000.

To our knowledge, existing nucleotide sequence analysis programs are not designed to analyze the spatial distribution of rare differences among very large sets of sequences representing thousands of species. We therefore created a set of analytic functions in Excel. For each position, the fraction of sequences with each nucleotide or amino acid residue was calculated and recorded in a frequency matrix. The most abundant (1^st^ modal) and second most abundant (2^nd^ modal) nucleotide or amino acid at each position, and the fraction of sequences occupied by these residues were determined, excluding sites with missing data. For each BARCODE, the number of sites that differed from the 1^st^ modal nucleotide and amino acid sequences was calculated. The sequence alignments and frequency matrices are available in Datasets S1 and S2, respectively. Sequences containing very low frequency variants (VLFs), defined as nucleotide or amino acid residues present in less than 0.1% of the set, were sorted according to whether the VLFs were present in single or multiple individuals of a species. Trace files archived in BOLD were examined in some cases as detailed below.

## Results

**Figure 1:**
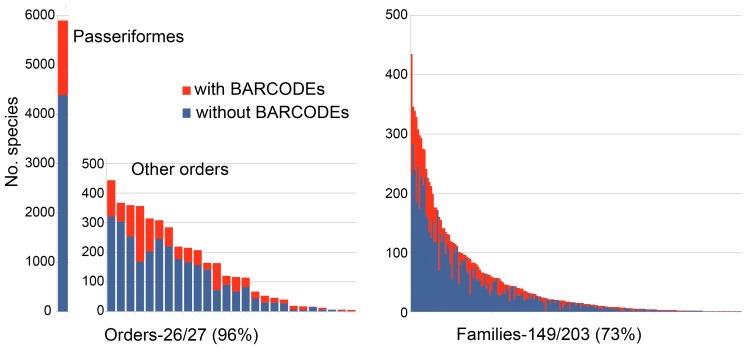
Representation of avian orders and families in BARCODE library.

To date GenBank contains about 16,000 COI sequences from 3,500 bird species. For this study, those with BARCODE keyword were analyzed. The avian BARCODE dataset comprised 11,333 records from 2,706 species, representing 27% of all bird species, 73% of families, and 96% of orders (Fig. 1). There were an average of 4.2 sequences/species (range 1–125); 573 species had single sequences.

**Figure 2:**
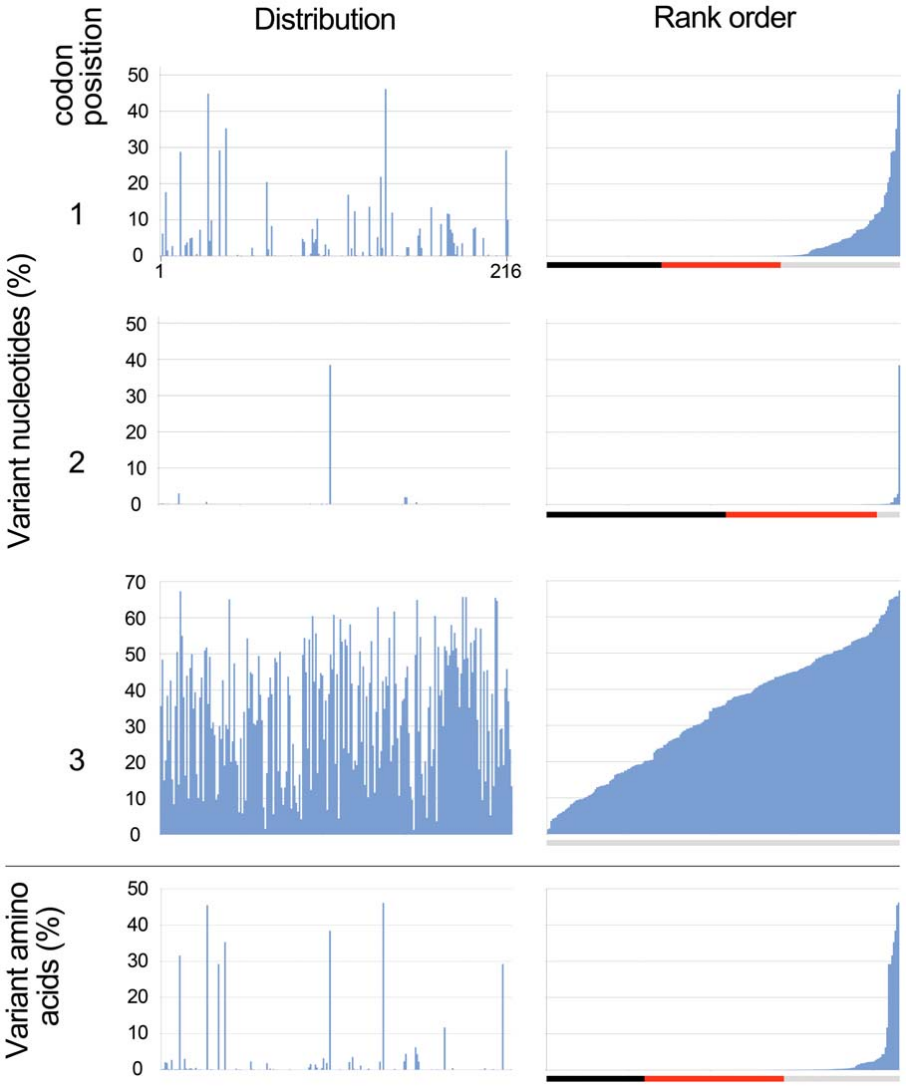
Variant nucleotide and amino acid positions among 11,333 avian BARCODEs. Bars below ranked histograms show conservation of positions: 100% (black), <100% and >99.9% (red), and <99.9% (gray).

Most nucleotide and amino acid positions in the COI barcode region were more than 99.9% conserved (Table 1, Fig. 2). Variation in the remaining sites was largely binary, i.e., limited to two of four nucleotides or two of 20 amino acids at a given position. As compared to the modal nucleotide and amino acid sequences, there was a relatively narrow range of variation, except at third codon positions (Table 1).

**Table 1:** Conservation of nucleotide and amino acid positions in avian BARCODEs.

	1^st^ modal 100%	1^st^ modal >99.9%	1^st^ +2^nd^ modal >99.9%	average no. differences vs. 1^st^ modal, range
Nucleotide 1^st^ codon position	68 (31)	141 (65)	198 (92)	5.6, 0–16
Nucleotide 2^nd^ codon position	110 (51)	202 (94)	216 (100)	0.5, 0–15
Nucleotide 3^rd^ codon position	0 (0)	0 (0)	70 (32)	72, 37–110
Amino acid	59 (27)	146 (69)	197 (97)	3.7, 0–15

Number of conserved and highly conserved positions and average number and range of differences from the 1^st^ modal sequence are shown. Percentages are given in parentheses.

### Distribution of very low frequency nucleotide variants

**Figure 3:**
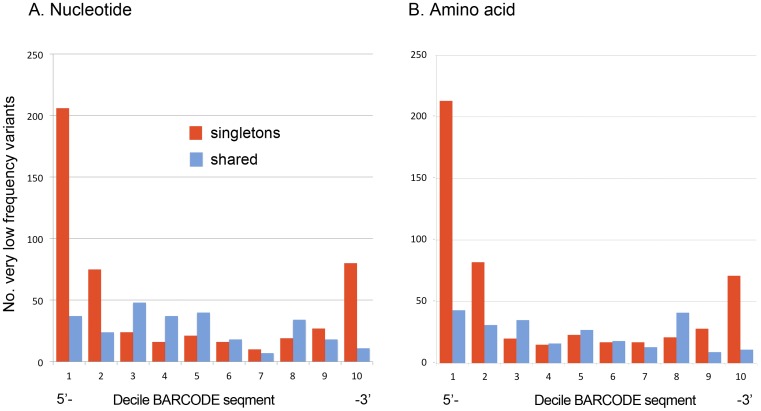
Distribution of very low frequency (VLF) variants across barcode segment. A) Nucleotide. B) Amino acid.

Sorting positions by variability demonstrated a long tail of nearly but not completely conserved sites (Fig. 2). To characterize further, BARCODEs containing very low frequency nucleotide variants (nVLFs) were selected for further analysis. nVLFs were categorized as to whether they were *singleton* variants present in one individual of a species, or *shared* variants present in two or more individuals of a species (Table 2). When analyzed by spatial location, singleton nVLFs were found to be strongly concentrated at the ends of the barcode segment, consistent with sequencing error (Fig. 3). In birds, the 5′ end of the barcode region is generally more difficult to sequence than the 3′ end, and the distribution of singletons matched this asymmetry. In contrast, shared nVLFs were relatively evenly distributed across the barcode segment, consistent with a biological origin (Fig. 3). Sliding window analysis is useful for detecting spatial patterns hidden in noisy data (e.g., [27]). Given the relatively simple patterns seen in Fig. 3, it was not surprising that a sliding window analysis also showed singleton but not shared nVLFs concentrated at the 5′>3′ ends of the barcode segment, mirroring the histogram distributions (Fig. S1).

**Table 2:** Singleton and shared very low frequency (VLF) variants in avian BARCODEs.

	Nucleotide			Amino acid			Concordance		
	VLFs	Seqs	Ave, range	VLFs	Seqs	Ave, range	nt only	aa only	both
Singleton	494	347	1.4, 1–15	507	391	1.3, 1–7	23	67	324
Shared	274	202	1.3, 1–5	244	190	1.3, 1–6	50	40	152
Pseudogene subset	40	13	3.1, 3–5	44	13	3.4, 2–6	0	0	13

The subset of shared VLFs contained in pseudogenes is shown at bottom. The concordance of sequences with nucleotide (nt) and amino acid (aa) VLFs is shown at right.

### Nucleotide sequencing error rate

Assuming the error rate is the same at all codon positions, it was possible to use the frequency of singleton nVLFs at second codon positions to calculate an error rate for the dataset. As nearly all (94%) second codon positions were >99.9% conserved, sequencing errors at these sites, if any, must be contained within the variable 0.1%, i.e., nVLFs. Possible “back mutation” errors at shared (biological) nVLF sites could be ignored, since such sites were present in only about 2% of sequences and therefore would make a negligible contribution to the total. BARCODEs representing a single individual of a species were excluded from the calculation, as it was not possible to determine if nVLFs in these sequences were shared among individuals of a species. Next, second codon position *shared* nVLFs were set aside as likely biological variants, leaving 187 second codon position singleton nVLFs scattered among the 10,760 BARCODEs that represented two or more individuals of species. Thus,


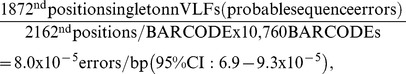


which is equivalent to approximately 0.05 errors/BARCODE (8.0 x 10^-5^ errors/bp x 648 bp/BARCODE).

As some singleton nVLFs may be unrecognized biological variants, this can be considered an upper limit for the true error rate.

### Very low frequency amino acid variants

It seemed likely that the rarity of nVLFs reflected strong selection against substitutions that result in amino acid changes. Thus it was not surprising that about 80% of nucleotide VLFs were associated with amino acid VLFs (aaVLFs), and vice versa, and that the distributions of singleton and shared amino acid and nucleotide VLFs across the barcode segment were similar (Table 2, Fig. 3).

Only four (0.2%) of the 2,133 species with multiple BARCODEs had three or more shared aaVLFs: *Nothoprocta ornata* (order Tinamiformes, family Tinamidae) (2 sequences, 6 shared VLFs), *Empidonax alnorum* (7 sequences, 2–3 shared VLFs) and *Cnemotriccus fuscatus* (2 sequences, 3 shared VLFs) (both in order Passeriformes, family Tyannidae), and *Branta canadensis* (order Anseriformes, family Anatidae) (2 sequences, 3 shared VLFs). These outliers might represent accurate COI sequences with an unusual number of rare substitutions, sequences with multiple errors, or overlooked pseudogenes. Trace files archived on Barcode of Life Data Systems (BOLD) were examined, as were all conspecific sequences including non-BARCODE records in GenBank. As detailed below, the outlier sequences appear to represent cryptic pseudogenes lacking stop codons.

For *N. ornata*, the VLF sequences (GenBank accession nos. JQ175579, JQ175580) were deeply divergent (12% Kimura 2-Parameter (K2P) distance) from a conspecific BARCODE (GenBank accession no. JQ175578) collected at the same locality. The BOLD ID engine gave similar results with an additional finding of five unpublished *N. ornata* records matching the non-VLF *N. ornata* BARCODE (Fig. S2). Finally, the *N. ornata* VLF BARCODEs had a 3 bp deletion at positions 619–621, confirmed by review of trace files, not found in any other of the 11,000 avian BARCODEs. Given these findings, we conclude that the *N. ornata* sequences with VLFs represent a pseudogene, overlooked due to the absence of stop codons and frameshift mutations.

**Figure 4:**
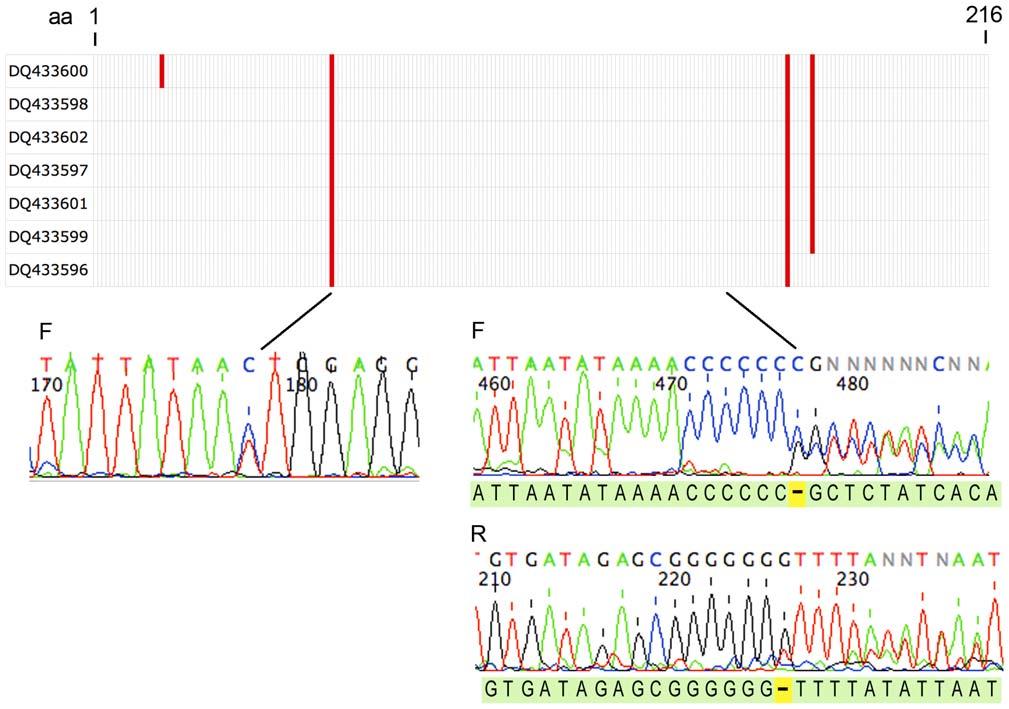
*Empidonax alnorum* BARCODEs represent a co-amplified pseudogene. Schematic shows positions of amino acid VLFs in barcode segment for *E. alnorum* BARCODEs with GenBank accession numbers as indicated. Representative trace files shown below display a double peak underlying the 5′ amino acid VLF and an overlooked single base insertion near nucleotide position 470. The GenBANK fasta file sequence corresponding to the insertion site is shown below the traces with the unrecorded nucleotide in yellow.

For *E. alnorum*, there were no conspecific sequences in GenBank without VLFs. However, trace files showed an overlooked single base insertion, which disrupts the reading frame, in a stretch of C's near position 470, followed by an abrupt transition to overlapping peaks downstream (Fig. 4). These findings are consistent with a pseudogene containing a single base insertion co-amplified with the native sequence. Upstream of insertion site, trace files show multiple double peaks including underlying the VLF at position 176, further evidence of co-amplification.

**Figure 5:**
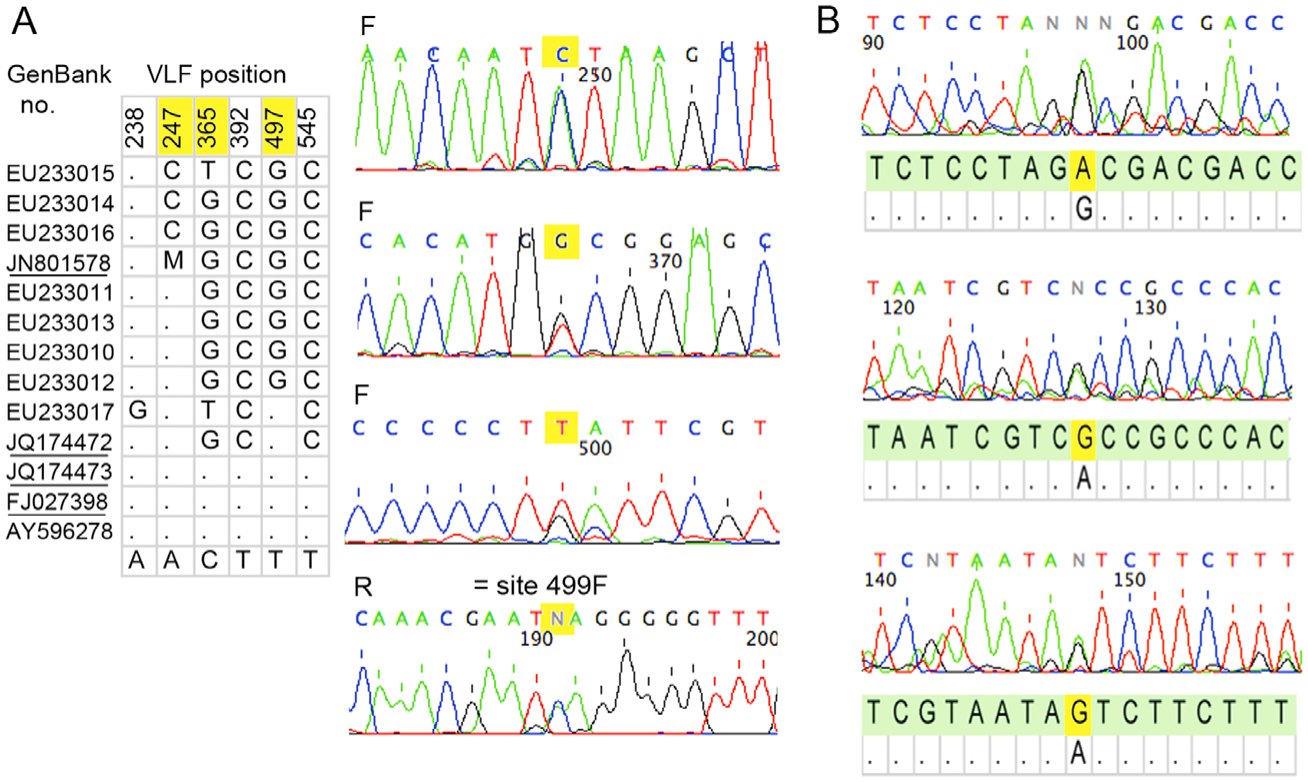
Additional BARCODEs with multiple shared amino acid VLFs reflect co-amplified pseudogenes. A) *Cnemotriccus fuscatus*. Nucleotides underlying amino acid VLFs for all *C. fuscatus* GenBank records are shown (underlined are BARCODEs); complete mitochondrial genome sequence AY596278 is at bottom. Representative trace files show double peaks at VLF positions highlighted in yellow (BARCODE numbering differs by 2 from forward trace files). B) *B. canadensis*. Trace files for outlier BARCODEs display double peaks at VLF sites. The GenBank fasta file sequence for these records is shown with the VLF nucleotide highlighted in yellow and the sequence from a representative conspecific BARCODE lacking VLFs below.

For *C. fuscatus*, the VLF sequences differed from conspecifics without VLFs by about 6% K2P. Trace files for outlier sequences showed multiple double peaks, including at three of the five VLF sites, consistent with a co-amplified pseudogene (Fig. 5A). For *B. canadensis*, there were multiple (>100) conspecifics without VLFs. Similar to the above cases, trace files for outlier sequences (DQ434449, DQ434453) showed double peaks at VLF sites consistent with co-amplification of a short pseudogene corresponding to the 5′ end of the barcode segment (Fig. 5B).

### Database quality comparison

**Figure 6:**
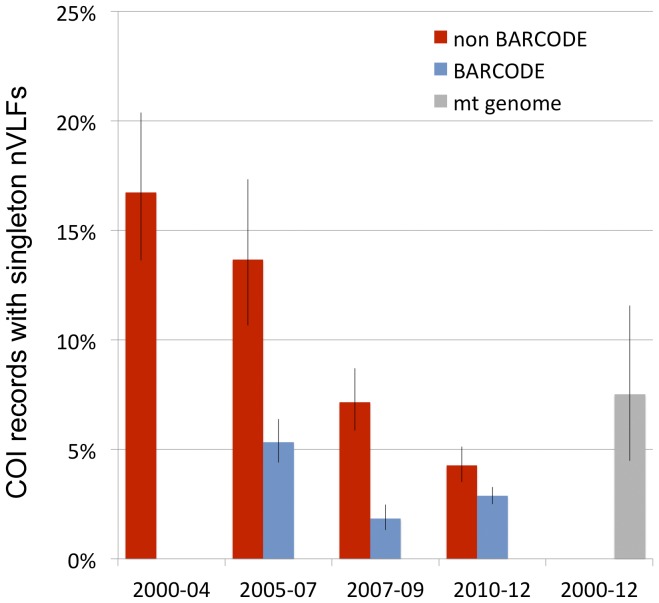
Prevalence of singleton nucleotide VLFs in avian BARCODE and non-BARCODE COI records deposited in GenBank since January 1, 2000. Intervals are January 1, 2000 to December 31, 2004 (2000–04); January 1, 2005 to June 30, 2007 (2005–07); July 1, 2007 to December 31, 2009 (2007–09), and January 1, 2010 to April 30, 2012 (2010–12). Bars indicate 95% confidence intervals.

We applied the nucleotide frequency matrix to determine differences among BARCODE vs. non-BARCODE records and changes over time. Sequences without conspecifics, previously published COI pseudogenes, and records labeled as “COI-like” were excluded. Barcodes extracted from complete mitochondrial genomes were analyzed separately. There were fewer sequences with singleton nVLFs, i.e., probable errors, among BARCODE as compared to non-BARCODE avian COI records, and significant improvement in both categories over the past decade (Fig. 6). COI sequences extracted from complete mitochondrial genomes had a greater prevalence of error than did recent BARCODE submissions.

## Discussion

In this study we applied frequency matrix analysis to 11,000 avian BARCODEs. We found that very low frequency variants present in single individuals of a species were strongly concentrated at the ends of the barcode segment, consistent with sequencing error. In addition, the frequency matrix approach led to recognizing a number of overlooked cryptic pseudogenes lacking stop codons. Our findings confirm the overall high quality of the avian dataset, supporting the effectiveness of BARCODE quality standards. The observed frequency of sequencing errors (on average about one error per 20 sequences) is unlikely to affect the accuracy of species identification.

**Figure 7:**
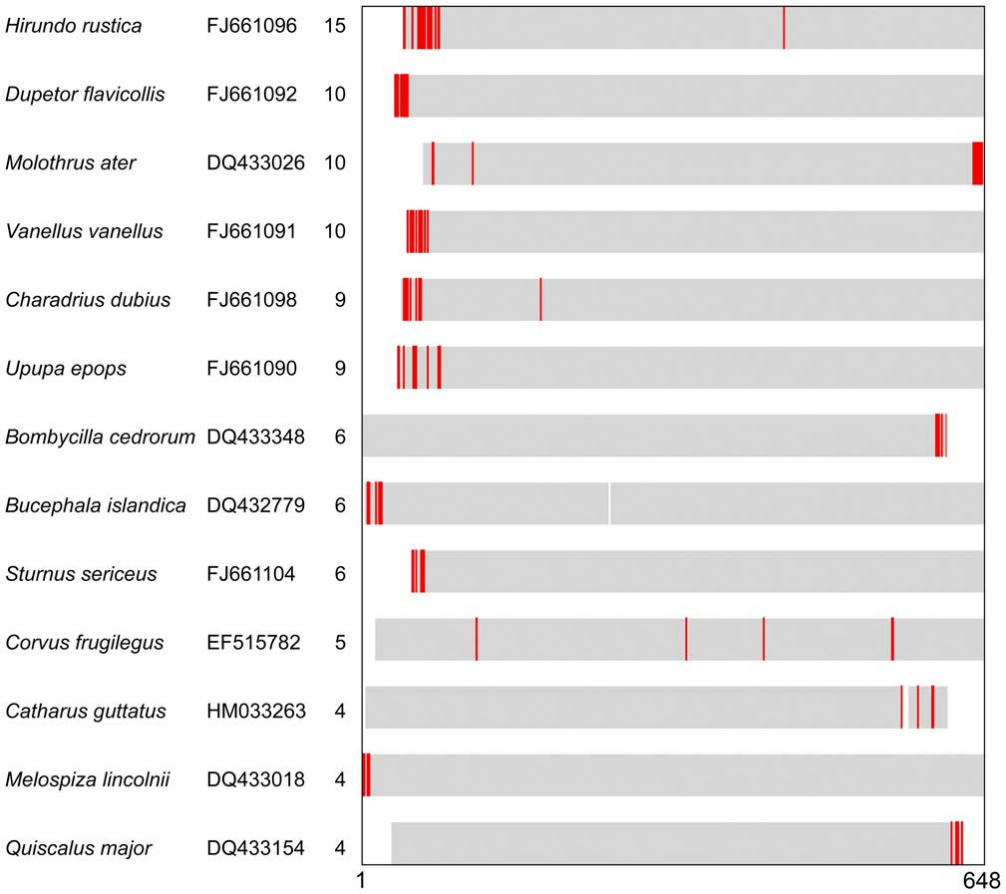
BARCODEs with four or more nucleotide singleton VLFs. Species name, GenBank accession number, and number of VLFs are shown. Gray bars indicate sequence alignment relative to 648 bp barcode region, VLFs are in red, and blanks indicate ambiguous positions. VLFs were concentrated at the 5′ or 3′ terminus, consistent with sequencing error, except for *C. frugilegus* EF515782, which appeared to be a pseudogene (Fig. S3).

The calculated upper limit for sequencing error, 8×10^−5^ errors/nucleotide, was relatively high for direct sequencing of PCR amplicons, the standard method for generating reference DNA barcodes [28], [29]. To our knowledge, this is the first estimate of sequencing error rate in a large BARCODE dataset created by multiple researchers. An advantage of the frequency matrix approach utilized here is that it flags probable errors directly, as opposed to an indirect indicator such as sequence quality. There are several limitations to the error rate calculation. First, a frequency matrix can only detect sequencing errors at positions that are otherwise highly conserved. At more variable positions, sequencing error is likely to result in a common biological variant. However, the error rate was based on singleton nVLFs at second codon positions, which were more than 99.9% conserved at nearly all (94%) sites, so this should not be a significant limitation. Second, some VLFs may be miscategorized. Although the set of singletons as a whole has a strongly U-shaped distribution indicating sequencing error, this may include accurate sequences with rare variants or pseudogenes (e.g., Figs. 7, S3). As above, we therefore consider this to be an upper limit of the true sequencing error rate.

We note that the error rate calculation is based on the observed pattern of variation in the set of avian BARCODEs examined. Other nucleotide sequence datasets including barcodes representing other groups may show reduced conservation at second positions, in which case this method of error rate calculation might not apply.

Pseudogenes are a recognized hazard to mitochondrial DNA analysis in general and DNA barcoding in particular [7], [30]–[35]. Most can be recognized by the presence of stop codons, insertions, deletions, or extreme divergence. However, cases with full open reading frames are described, including some that differ minimally from the mitochondrial sequence [30]. To date, eight avian COI pseudogene sequences with open reading frames are reported [32], [36]. When applied to the frequency matrix generated in this study, these contained 7–10 nucleotide and amino acid VLFs, strengthening the observation that pseudogenes can be identified by the presence of multiple VLFs. A similar approach, which measured deviation from a consensus sequence derived from a multi-species alignment, identified pseudogenes among a large family of human olfactory receptor genes and is the basis of a tree-building detection method using Pfam database alignments [37], [38]. As an extension of the present analysis, it may be useful to catalog the substitutions found in pseudogenes as compared to those in species with shared VLFs. Two of the four species flagged in this study and six of seven species with published pseudogene sequences are tyrannid flycatchers, which might reflect a limitation of standard barcode primers in this group. The publicly archived trace files demonstrated that co-amplification accounted for VLFs in pseudogene sequences, highlighting the importance of this component of the BARCODE data standard [4].

For sequences identified as pseudogenes there is enough evidence to justify revising the GenBank records including removal of the BARCODE keyword. For records containing probable sequencing errors, there is no established way to incorporate this sort of information. Annotating sequence files in GenBank or BOLD might be useful, particularly for those with multiple VLFs (Fig. 7). On a practical level, one or two errors in 648 bp barcode, equivalent to 0.15–0.30% K2P distance, are unlikely to result in an error in species identification given that most closely related animal species differ by 2% or more, although there are numerous exceptions to this rule including several among birds [6], [8], [39], [40]. Even a much larger number of errors may not affect assignment unless they happen to involve diagnostic sites that differ among closely-related taxa. This supposition is supported by observation that BARCODEs with the largest number of probable errors (Fig. 7) nonetheless gave closest matches with >98% identity to conspecific sequences in GenBank using BLAST.

The finding that most very low frequency residues in this dataset are probable sequencing errors or contained in pseudogenes may be important for studies of rare variants, including population biology, RNA editing, and somatic mutation [41]–[45]. Errors in cloned PCR products are known stumbling blocks; present results suggest this caution extends to directly sequenced products as well. Although the avian BARCODE data add to the observation that rare variants in animal mitochondrial DNA are largely missense substitutions, most of what appeared to be mutations were in fact errors, suggesting careful reexamination of prior studies [46]–[49]. A combined frequency matrix-spatial analysis approach may also be useful for evaluating newer technologies such as pyrosequencing which have the potential to generate enormous numbers of sequences. It is recently reported that up to 94% of putative RNA editing events reflect machine errors near the ends of pyrosequencing reads [50], [51].

Our results strengthen the evidence for tight functional constraint on COI [52]–[55]. Most of the variation that does occur is limited to two nucleotides or amino acids at a position. We note that only eight amino acid positions differ in more than 5% of sequences in this dataset (Fig. 2). It may be of interest to determine whether these are associated with taxonomic groups or whether there is toggling back and forth which could underlie some of the difficulties in evolutionary inference using mitochondrial sequences [56]. There were about 60 species with one or two shared amino acid VLFs. These may represent taxa that are poorly represented in the dataset, sequencing errors shared among conspecifics, overlooked pseudogenes, or interesting exceptions harboring what otherwise appear to be prohibited variants.

As widely observed in protein coding genes in general and COI in particular, the degree of conservation differed by codon position, with 2^nd^ >1^st^ >>3^rd^ [57], [58]. For this dataset of 11,000 records, the calculated ratio of variance was 1: 11: 146. In addition to magnitude differences, the distribution of variation also differed among codon positions: strongly curved at 1^st^ position and nearly linear at 3^rd^ position (compare rank ordered panels in Fig. 2). Modeling might help understand how evolutionary diversification leads to different patterns of variance by codon position.

In this study we show that a frequency matrix can be applied to quantify errors in avian BARCODEs. We identified probable sequencing errors and pseudogenes, information that can be used to improve what is already a high quality database. To test whether this approach is useful for other barcode datasets, the analysis could be extended to fish (Actinopterygii: 20,000 BARCODEs, 3,500 species) and moths and butterflies (Lepidoptera: 170,000 BARCODEs, 21,000 species). In addition to evaluating BARCODEs, the frequency matrix approach described here may have general utility as a method for identifying errors and flagging pseudogenes in other large, multi-species sequence datasets containing highly conserved residues.

## Supporting Information

Figure S1 **Sliding window analysis (window = 30 nucleotides) of singleton and shared nucleotide VLFs in avian BARCODEs.**
(TIF)

Fiure S2 **BOLD ID Tree for** ***Nothoprocta ornata*** **BARCODE JQ175578.** The query BARCODE with no VLFs shown in red matches itself and five unpublished *N. ornata* records and is distant from the *N. ornata* BARCODEs with six VLFs.
(TIF)

Figure S3 ***Corvus frugilegus*** **BARCODE EF515782 is a pseudogene.** A) K2P NJ Tree for all GenBank *C. frugilegus* COI sequences, with BARCODE EF515782 highlighted in yellow and number of aaVLFs in parentheses. B) Trace files for *C. frugilegus* BARCODE EF515782 showing double peaks underlying VLF sites highlighted in yellow.
(TIF)

Dataset S1 **Aligned nucleotide sequences of avian BARCODES in fas format.**
(FAS)

Dataset S2 **Nucleotide and amino acid frequency matrices and modal sequences generated from avian BARCODE dataset.**
(XLSX)
