# Supplementary figures and images for: Frequency Matrix Approach Demonstrates High Sequence Quality in Avian BARCODEs and Highlights Cryptic Pseudogenes
Source: PLoS One. 2012 Aug 27;7(8):e43992. doi: 10.1371/journal.pone.0043992 (PMC3428349; doi:10.1371/journal.pone.0043992)

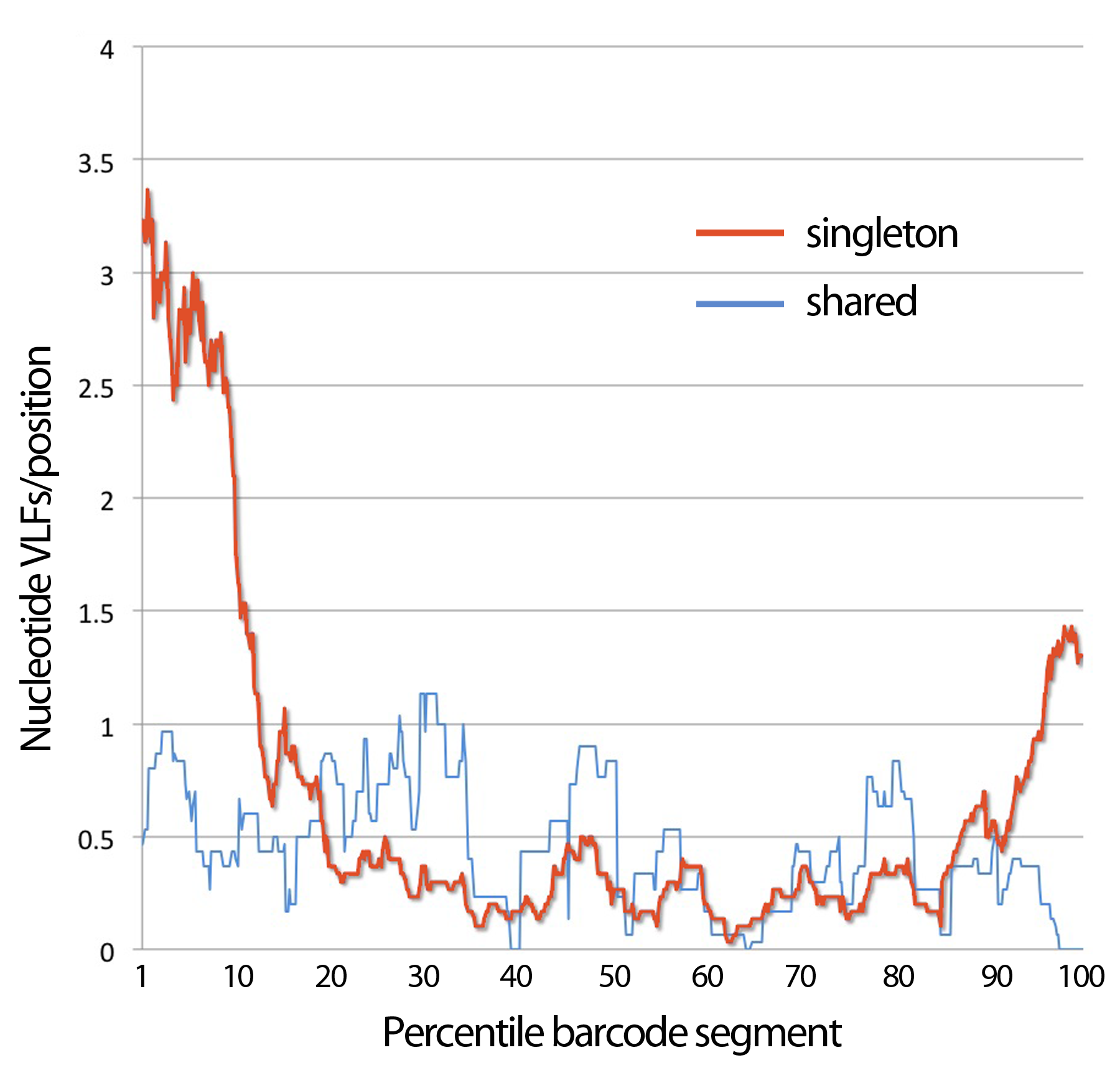

Supplement: Figure S1 — Sliding window analysis (window = 30 nucleotides) of singleton and shared nucleotide VLFs in avian BARCODEs. (TIF) [file pone.0043992.s001.tif]

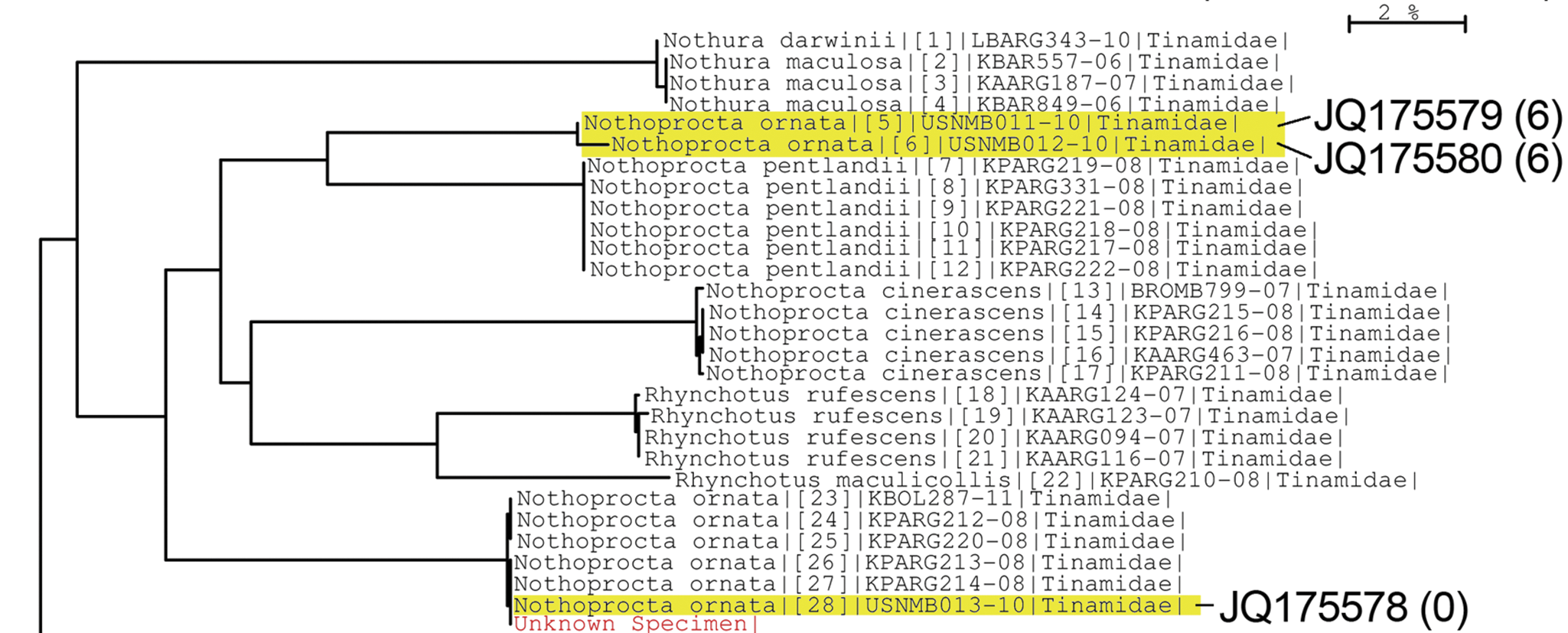

Supplement: Fiure S2 — BOLD ID Tree for Nothoprocta ornata BARCODE JQ175578. The query BARCODE with no VLFs shown in red matches itself and five unpublished N. ornata records and is distant from the N. ornata BARCODEs with six VLFs. (TIF) [file pone.0043992.s002.tif]

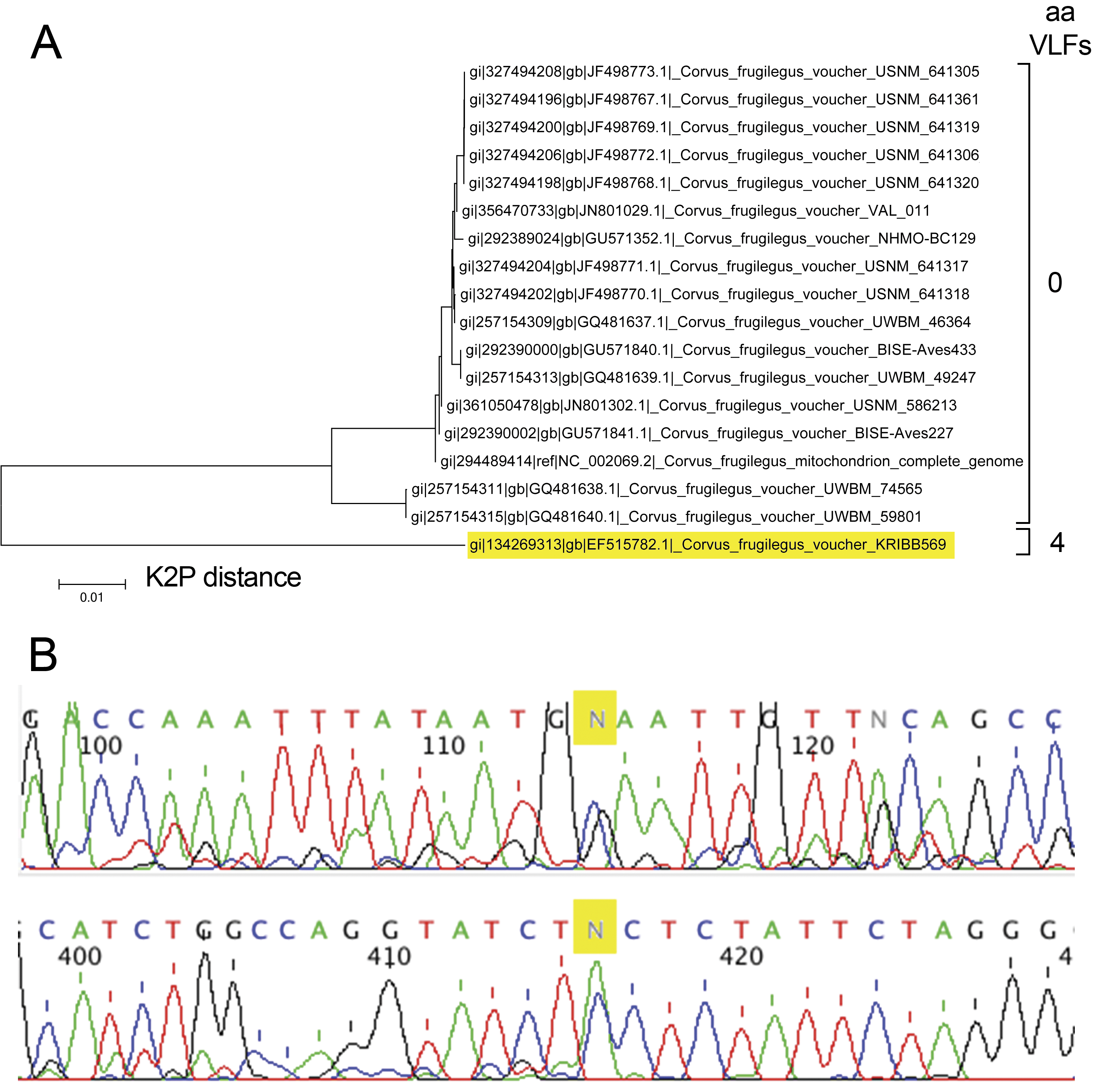

Supplement: Figure S3 — Corvus frugilegus BARCODE EF515782 is a pseudogene. A) K2P NJ Tree for all GenBank C. frugilegus COI sequences, with BARCODE EF515782 highlighted in yellow and number of aaVLFs in parentheses. B) Trace files for C. frugilegus BARCODE EF515782 showing double peaks underlying VLF sites highlighted in yellow. (TIF) [file pone.0043992.s003.tif]
